# Evaluation of adefovir & lamivudine in chronic hepatitis B: Correlation with HBV viral kinetic, hepatic-necro inflammation & fibrosis

**Published:** 2011-01

**Authors:** Kumar S Pradeep, Subhash Medhi, Mohammad Asim, Bhudev C Das, Ranjana Gondal, Premashis Kar

**Affiliations:** *Department of Medicine, Maulana Azad Medical College, New Delhi, India*; * *Department of Medicine, Ambedkar Centre for Biomedical Research, University of Delhi, Delhi India*; ** *Department of Pathology, G.B. Pant Hospital, New Delhi, India*

**Keywords:** Adefovir, chronic hepatitis B, HBV DNA, lamivudine

## Abstract

**Background & objectives:**

Chronic hepatitis B is an important cause of morbidity and mortality. We conducted a study comparing the efficacy of adefovir and lamivudine with respect to their impact on serum and hepatic viral DNA clearance, and improvement in hepatic necro-inflammatory score, in naive patients of chronic hepatitis B.

**Methods:**

This prospective randomized pilot study was conducted in Lok Nayak Hospital, New Delhi, involving 30 patients of chronic hepatitis B (both e antigen positive and negative); 15 were randomly selected to receive either adefovir or lamivudine for a period of 6 months. Quantification of serum and hepatic HBV DNA levels was done by real time PCR and liver biopsy was done at the beginning and end of 6 months.

**Results:**

Serum ALT was elevated to 2 or more times normalized in both the groups. In the adefovir group, two patients became HBeAg negative. In the lamivudine group, one patient became HBeAg negative. After therapy HBV DNA was negative in 26.7 per cent patients from adefovir group and 13.3 per cent patients from lamivudine group. Serum HBV DNA levels were correlated with the hepatic levels before therapy (r=0.843; P<0.001) and after therapy (r=0.713, P<0.001) showing strong correlation. There was a median reduction of 1.92 and 2.06 log copies per ml in serum HBV DNA load after adefovir and lamivudine therapy, respectively. The mean reduction in the histotogy activity index (HAI) score was 2 and 1.53, fibrosis score was 2.33 and 3.06 after adefovir and lamivudine therapy respectively.

**Interpretation & conclusions:**

Adefovir and lamivudine treatment caused biochemical and serological improvement when administered for about 6 months with significant reduction in HBV DNA, serum and hepatic viral load without completely clearing the virus from either serum or liver. It also helped in reduction of the necro-inflammatory and fibrosis score of patients with chronic hepatitis B. Our study also showed significant correlation between serum and hepatic HBV DNA levels both before and after therapy. There was not enough evidence to show therapeutic advantage of one drug over the other in any of the parameters measured.

Viral hepatitis is a disease of antiquity which is caused by five primary hepatotropic human pathogenic viruses namely A, B, C, D and E^1^. Hepatitis B virus (HBV) is probably the second most common cause of viral hepatitis (after A-like non-A non-B), and it is probably the most common cause of chronic liver disease, and the infected individuals are at an increased risk to develop cirrhosis, hepatic decompensation and hepatocellular carcinoma (HCC)^2^,^3^. The average estimated carrier rate of HBV in the world is 5 per cent (400 million people), about 4 per cent in India, with a total pool of approximately 36 million carriers^4^. Highest prevalence has been reported amongst the aborigines of Andaman and Arunachal Pradesh^5^,^6^. The outcome of these infections is either an asymptomatic disease or acute symptomatic hepatitis B which is self limited in 90 per cent of cases. Patients may become immune to HBV or may develop a chronic carrier state in 10 per cent of cases, which is generally life-long^7^. This carriage may occur either after acute hepatitis or after an asymptomatic form and chronic infection with HBV can be either “replicative” or “non-replicative”. Therapeutic advances help lower viral load in chronic hepatitis B patients and this has been shown to have significant impact on the incidence of liver-related morbidity and mortality. Interferon has been the only therapeutic choice for more than 20 years. However, sustained suppression of viral replication and resolution of hepatitis B is achieved in only about one third of patients and treatment is associated with considerable side effects^8^. With the advent of therapeutic agents initially designed for human immunodeficiency virus infection, new treatment options have become available. Lamivudine, a second-generation nucleoside analogue, efficiently inhibits HBV replication with an improvement of liver histology in most patients. However, lamivudine resistance due to mutations in the viral polymerase gene is frequent^9^. More recently, the nucleotide analogue adefovir dipivoxil, the prodrug of adefovir, was licensed for treatment of chronic hepatitis B^10^. Clinical efficacy parallels that of lamivudine in hepatitis B e antigen- positive patients. Lamivudine-resistant HBV variants are sensitive to adefovir action and, remarkably, no mutations conferring viral resistance to adefovir occurred during the 48-week treatment course reported in two initial studies^10^,^11^.

There are no studies to correlate hepatitis B viral kinetics with the therapeutic response of the drugs particularly adefovir and lamivudine. The Indian data on these aspects have been very limited. Therefore, we conducted a study in chronic hepatitis patients to compare adefovir and lamivudine where the drug efficacy was evaluated with hepatic HBV DNA clearance as the end point in serum and compared that of HBV DNA in the liver, and necro-inflammatory changes in liver tissue at base line and after 6 months of therapy.

## Materials & Methods

### 

#### Patients:

The study group consisted of all consecutive chronic hepatitis patients who were diagnosed as per AASLD (American Association for the Study of Liver Diseases) practise guideline^12^ from the out patient department and medical wards of Lok Nayak Hospital, New Delhi, during the period January 2007 to December 2008. The patients were naïve for antiviral therapy against hepatitis B within 24 wk before randomization, had detectable hepatitis B surface antigen (HBsAg) for more than 6 months, serum HBV DNA > 10^5^ /ml, persistent or intermittent elevation in alonine aminotransferase/aspartate aminotransferase (ALT/AST) levels(≥2), liver biopsy showing chronic hepatitis and negative urine or serum pregnancy test (for women of childbearing potential). All fertile men with partners of childbearing age and premenopausal women were required to use reliable contraception during the study and for six months after treatment completion.

Patients were excluded at screening if they had been treated previously with interferon or had received antiviral or immunosuppressive medications; tested positive for antibody to hepatitis C virus (HCV), hepatitis D virus (HDV) or human immunodeficiency virus (HIV) and for pregnancy; had decompensated liver disease; had a medical condition associated with chronic liver disease other than viral hepatitis; and alcohol and/or drug abuse within one year of study entry. All patients and their relatives signed the informed consent form before enrollment. The study was approved by the local ethical committee of Maulana Azad Medical College, New Delhi.

#### Procedure:

Serum samples were collected from patients (5 ml) and stored at -70° C. Test for liver functions, serological tests for HBsAg (Ranbaxy Diagnostics, England), anti-HBe (General biological, Taiwan), HBeAg (General biological, Taiwan), Anti-HbcIgG (Radim diagnostic, Rome, Italy), HBV DNA and HBV viral load were assessed at base line for inclusion of patients. Ultrasonography (USG) of the abdomen was also done to look for liver size and echotexture, spleen size portal vein in indicated patients. The patients were randomly assigned to receive either Hepsara (adefovir-10 mg) or Lamivir (lamivudine-100 mg) daily for a period of 6 months (provided free from Ranbaxy). At the end of 6 months of therapy, those who continued to have a high viral DNA load continued to receive therapy for a period of one year. Those who became e antigen negative or DNA negative with either of the therapy were kept on follow up to ascertain sustained virological response.

#### Histological analysis:

Liver biopsy was done in all patients to assess severity of disease before the start of therapy and at the end of 6 months and assessed by a single independent histopathologist who was blinded with respect to patient identity, treatment assignment, laboratory data, and date of biopsy specimen. The biopsy specimens were scored according to the original criteria of Knodell histological activity index (HAI)^13^.

#### Extraction of DNA from serum & polymerase chain reaction (PCR):

HBV DNA was extracted from serum by using the standard phenol chloroform method. Detection of HBV DNA was carried out using primers as described earlier with slight modification^14^ to amplify part of the surface gene (nucleotide position 425 to position 840). PCR was carried out in 0.5 ml reaction tube, each containing 6.7 mM MgCl_2_, all four deoxynucleotides at 1.1 mM each, 20 pmol of each primer, and 1U of Thermus aquaticus polymerase and 2 µl of sample DNA in a final volume of 50 µl. PCR was done in a 50 µl reaction mixture in thermocycler and amplification reaction carried out in 40 cycles at 94° C for 1 min, 53° C for 1 min and 72° C for 1 min, with a 10 min extension step at 72° C at the end; 10 µl of the reaction mixture was analysed by electrophoresis in 2 per cent agarose gel that was stained with ethidium bromide and examined under UV light to detect an amplicon size of 416 base pair.

#### Viral load estimation of hepatitis B, by real time PCR:

Hepatitis B viral load estimations was performed by real time PCR (RT-PCR) method with the help of a Light Cycler instrument (Corbet Research, Australia) using Geno Sen’s HBV Real time PCR kit (Genome Diagnostic, India) according to the manufacturer’s instruction. The number of viral copies per ml was estimated against different standards.

#### Statistical analysis:

Statistical analysis was performed using SPSS (Statistical Package for Social Sciences), for Windows, release 11.00., standard version, (copyright SPSS Inc., 2001). Qualitative values were correlated with Chi-square or Fisher exact tests. *P* < 0.05 (two-tailed) was considered statistically significant. HBV DNA levels are expressed as logarithmic scales. Quantitative values are expressed as means and ranges, and were compared using the Student’s t-test or the Mann- Whitney non-parametric *U* test. Kaplan-Meier estimates and log-rank analyses were used to identify factors associated with the time to HBeAg seroconversion and test for the comparison of median values of two independent groups.

## Results

A total of 670 patients were screened during the study period, of whom 30 patients were selected and randomized in 1: 1 ratio for the lamivudine and adefovir arm after fulfilling the inclusion and exclusion criteria. For the histological assessments, paired liver biopsies were evaluated in both the group. Both the treatments groups were compared with respect to baseline demographics and disease characteristics (Tables I, II). Overall, there were 21 males and 9 females and the mean age was 27.53 ± 8.67 (18-60 yr). The two groups were comparable with respect to age and sex distribution. All the patients in both the groups completed the 24 wk of therapy.

**Table I:** Characteristics of patients with chronic hepatitis B sequentially treated with lamivudine and adefovir

Characteristic	Lamivudine group (N=15)	Adefovir group (N=15)	*P* value
*Age*(yr)	25.53 ± 9.23	29.53 ± 6.67	NS
Median (range)	38 (18-70)	40 (20-72)	0.9
Mean ± SD	29.73 ± 10.06	25.33 ± 6.64	0.7
Sex (male/female)	12/3	9/6	0.2
*Serum alanine aminotransferase*(IU/l)			
Baseline Median (range Mean ± SD	85 (80-109) 93 ± 8.5	84 (74-100) 88.6	0.0001
End of treatment Median (range Mean ± SD	26 (20-37) 27.2 ± 5.26	28 (13-44 28.13 ± 8.66	
*Positive for HBeAg (%)*	87	87	-
*Serum bilirubin (mg/dl)*			
Mean ± SD	0.73 ± 0.33	0.77 ± 0.25	0.89
Median (range)	0.7 (0.2-1.3)	0.6 (0.4-1.2)	-
*Histologic activity index score*			
Baseline Median (range Mean ± SD	3 (2-6 2.8 ± 1.01	4 (2-9) 3.53 ± 1.92	0.53
End of treatment Median (range Mean ± SD	1 (1-2) 1.27 ± 0.45	2 (1-3 1.53 ± 0.74	0.6
*Fibrosis score*			
Baseline Median (range Mean ± SD	3 (2-6) 2.8 ± 1.01	5 (2-9 3.53 ± 1.92	0.53
End of treatment Median (range Mean ± SD	1 (0-1 0.47 ± 0.5	1 (0-3) 0.47 ± 0.8	-
*Serum HBVDNA (copies/ml) Median (range)*			
Baseline Post-follow up	8.3 × 10^10^ - 8.7 × 10^5^(5.6 × 10^9^ ± 2.1 × 10^10^ 9.8 × 10^6^- 890 (1.4 × 10^6^ ± 2.6 ×10^6^)	6.7 × 10^10^-3.3 × 10^5^(4.8 × 10^9^ ± 1.7 × 10^10^) 8.1 × 10^7^- 258 (8.9 × 10^6^ ± 2.1 × 10^7^)	0.001
*Hepatic HBV DNA*(copies/ml) Median (range)			
Baseline Post-follow up	9 × 10^7^ - 789 (7.6 × 10^6^ ± 1.3 × 10^7^ 9.1 × 10^4^- 831 (2.2 × 10^4^ ± 3.5 ×10^4^)	4.1 × 10^7^-6.2 × 10^3^ (7.6 × 10^6^ ± 1.3 × 10^7^ 9.3 × 10^6^- 91 (1.1 × 10^6^ ± 2.7 × 10^6^)	0.001

**Table II:** Loss of HBeAg, serum HBV DNA and ALT elevations after therapy

Characteristic	Lamivudine group (N=15)	Adefovir group (N=15)
Seroconversion of HBeAg	1	2
Loss of HBV DNA	2	4
Loss of HBsAg	0	0
Insrease in ALT levels (>2X ULN)	0	0

There was a significant reduction in the serum ALT levels in both the adefovir and the lamivudine groups after treatment for 6 months (*P*<0.0001) (Table I). There was no significant difference in the total serum bilirubin, serum albumin, ALP and prothrombin time before and after treatment in both the groups. The blood urea, serum creatinine remained normal at the end of 6 months of treatment.

All the patients remained HBsAg positive at the end of 6 months in both the groups. In the adefovir group two (15.38%) patients seroconverted from HBeAg positive to negative state. In the lamivudine group, one (7.7%) patients seroconverted from HBeAg positive status to negative status.

Lamivudine and adefovir were well tolerated, with no incidence of adverse events during the 24 wk therapy. There were no deaths or instances of hepatic decompensation except in one patient in the adefovir group who developed cirrhosis diagnosed by USG abdomen, although it remained asymptomatic. The clinical laboratory value was similar in the two study groups (Table I). During therapy, similar proportions of both groups had elevations in serum alanine aminotransferase. There was a significant reduction in the necro-inflammatory and fibrosis scores in both the groups (*P*<0.001) (Table I).

**Fig. 1:**
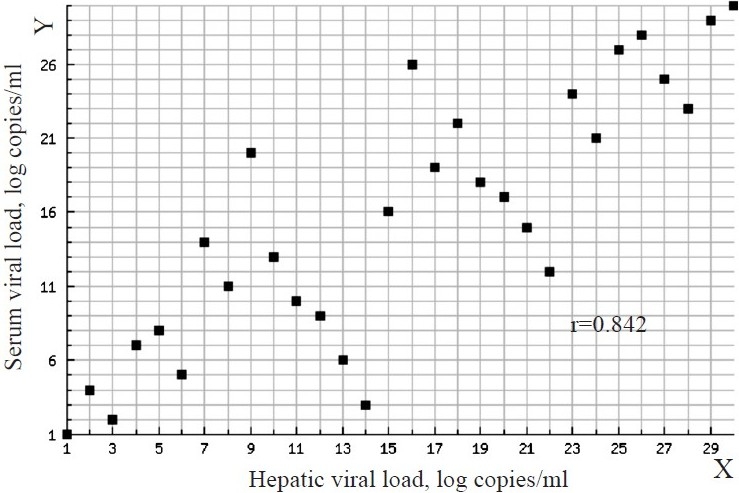
Correlation between serum viral load (Y-axis) and hepatic viral load (X-axis) at baseline. Spearman’s rank correlation coefficient – r: 0.842 between serum viral load and hepatic viral load at base line.

**Fig. 2:**
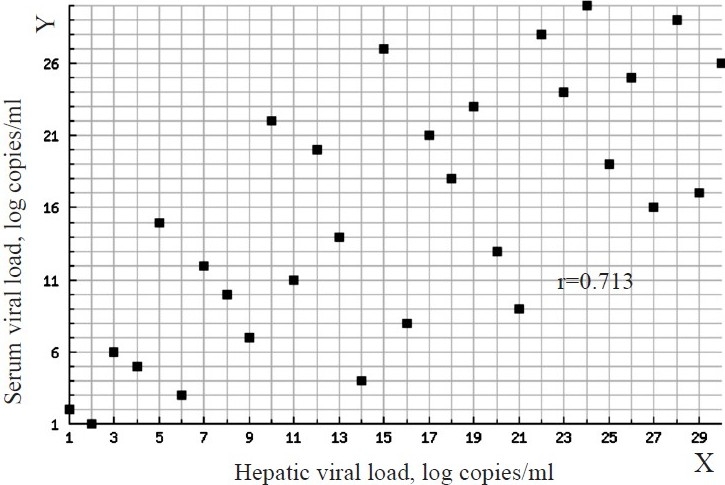
Correlation between serum viral load (Y-axis) and hepatic viral load (X-axis) after treatment. Spearman’s rank correlation coefficient – r: 0.713 between serum viral load and hepatic viral load after treatment.

In adefovir group, four (26.7%) patients became HBV DNA negative after 6 months of therapy. In the lamivudine group, two (13.3%) patients became HBV DNA negative. In both the groups, there was a significant reduction in the serum and hepatic HBV DNA load after 6 months of therapy (*P*<0.001). At the baseline and at the end of therapy there was a significant correlation between the serum and hepatic HBV DNA levels (Figs 1 and 2) Individually, baseline correlation in the adefovir group showed r=0.836 and lamivudine group, r=0.768 (*P*<0.001) Similar results were seen after 6 months of therapy, in adefovir group r =0.70 and in the lamivudine group r=0.682 (*P*<0.004 and 0.005 respectively).

The mean reduction in the HAI score was 2 and 1.53 in adefovir and lamivudine group but statistically it was not significant. The mean reduction in fibrosis score was 2.33 and 3.06 in adefovir and lamivudine group which was also not significant. There was a median reduction of 1.92 log copies per ml and 2.06 log copies per ml in serum HBV DNA load after adefovir therapy and lamivudine therapy respectively (Table I).

## Discussion

Both HBeAg-positive and HBeAg-negative infection can potentially result in progressive liver disease and affect about 350 million people worldwide^3^. The goal of antiviral therapy is to arrest or delay the progressive HBV-associated hepatic injury, and histologial improvements are thought to be effected by the suppression of viral replication. The present study showed that both adefovir as well as lamivudine brought about early normalization of serum ALT levels with reduction in serum and hepatic HBV DNA load, also reduction in the HAI and fibrosis scores in patients with chronic hepatitis B. But there was no complete clearance from liver, showing that liver still harboured infection, raising the possibility of re-activation.

Adefovir was given to 15 patients of chronic hepatitis B for 6 months. There was normalization of serum AST levels in all the patients (100% improvement) with a median reduction of 64 IU/l. Other studies have reported median reductions of 59 IU/l in serum ALT levels at week 96 and normalization in 48-73 per cent of patients and HBeAg seroconversion in 12 per cent^11^,^15^. HBV DNA was negative in 26.7 per cent patients after therapy. Other studies with HBeAg positive patients reported that at the end of 24 and 52 wk, mean HBV DNA reduction was 4.97 and 4.00 log copies/ml respectively^16^ and 3.47 log copies/ml at 96 wk^17^. In our study, there was a median reduction of 1.92 log copies/ml in serum HBV DNA load after 24 wk therapy, which was statistically significant. This might be due to less severe disease stage as diagnosed by liver histology and USG at baseline. In this study there was 100 per cent histological improvement whereas Marcellin *et al*^11^ reported 53 per cent histological improvement.

In the lamivudine group there was normalization of serum AST levels in all patients (100% improvement) with a median reduction of 68 IU/l. In this group, one patient seroconverted to HBeAg negative status, two were negative at the baseline and continued to be HBeAg negative which was comparable with the study by Yao *et al*^18^ in which there were 9.5 per cent HBeAg loss. In other studies it has been reported that 32 per cent of the patients became HBeAg negative and normalization of serum ALT levels was in 41 - 81.8 per cent of the cases after 52 wk therapy^18^,^19^. HBV DNA became negative in only 13.3 per cent patients after lamivudine therapy whereas ealier studies have reported undetectable HBV DNA levels in 45-98 per cent of patients at 52 wk^18^,^19^,^20^. In patients who are treated with interferon, sustained reductions in serum HBV DNA levels occur primarily in those with HBeAg responses. In contrast, in our study, substantial reductions in serum HBV DNA levels occurred in virtually all lamivudine recipients, independent of the HBeAg responses. In a study by Lai *et al*^21^, at the end of 52 wk of therapy, hepatic necro-inflammatory activity improved by 2 points or more in 56 per cent of patients receiving 100 mg of lamivudine and worsened in 7 per cent. It was associated with reduced progression of fibrosis and 16 per cent HBeAg seroconversion. There was 98 per cent reduction in the HBV DNA load compared to the baseline, and 72 per cent normalization of ALT. Similarly in a study by Manolakopoulos *et al*^22^, initial virologic and biochemical response was observed in 92 and 89 per cent patients, respectively indicating that high levels of necro-inflammation in liver biopsy were associated with a higher probability of initial virological and biochemical response.

Our study should a strong correlation between serum and hepatic HBV DNA levels which corroborated with other’s findings^23^,^24^.

There have been reports of occasional, severe elevations in serum alanine aminotransferase after treatment^25^ ; in our study, however, these elevations were not clinically severe and were not associated with hepatic decompensation. Preliminary data suggest that long-term therapy is well tolerated^26^ ; studies to assess the effects of long-term suppressive therapy in patients who do not seroconvert are in progress. It is not certain whether the results of treatment with a combination of lamivudine and adefovir would be better than the results of treatment with either drug alone. On the basis of initial reports from controlled studies, combination therapy does not appear to have greater benefit than lamivudine or adefovir monotherapy^27^.

Our study had certain limitations of having a small sample size; also the treatment was only for 6 months, which was not sufficient to bring about certain changes like HBsAg seroconversion and also total clearance of DNA from the serum.

In summary both adefovir and lamivudine treatment led to virological, serological and biochemical improvement in patients with a chronic hepatitis B with a decrease of histological index although it does not show a strong correlation with the viral clearance after 6 month of treatment as none of the patients showed complete clearance of viral DNA.
